# Radiopharmaceuticals in Malignant Melanoma: A Comprehensive Review of Diagnostic, Therapeutic, and Immune-Related Applications by PET/CT, SPECT/CT, and PET/MRI

**DOI:** 10.3390/diagnostics15182305

**Published:** 2025-09-11

**Authors:** Irina Pirsan, Doina Piciu

**Affiliations:** 1 Nuclear Medicine Department, University Emergency Hospital Bucharest, 050098 Bucharest, Romania; 2 Doctoral School, University of Medicine and Pharmacy “Iuliu Hatieganu”, 400012 Cluj-Napoca, Romania; doina.piciu@umfcluj.ro; 3 Affidea Cluj-Napoca, 400487 Cluj-Napoca, Romania

**Keywords:** malignant melanoma, molecular imaging, PET/CT, PET/MRI, SPECT/CT, radiotracers, theranostics, immune-related adverse events

## Abstract

**Background:** Malignant melanoma remains an oncological challenge, with advanced-stage five-year survival rates under 20%. Precise molecular imaging has become indispensable for accurate staging, selection of targeted or immunotherapies, treatment response assessment, and early detection of immune-related adverse events. This review examines the roles of PET/CT, PET/MRI, and SPECT/CT radiopharmaceuticals in melanoma management and highlights novel tracers and theranostic strategies poised to enhance precision nuclear medicine in this disease. **Methods:** We performed a review of English-language literature from January 2000 through June 2025, querying PubMed, Scopus, and clinical-trial registries for original research articles, meta-analyses, clinical guidelines, and illustrative case reports. Eligible studies investigated PET/CT, PET/MRI, or SPECT/CT applications in melanoma diagnosis, nodal and distant staging, therapy monitoring, irAE (immune-related adverse events) detection, and the development of emerging radiotracers or theranostic radiopharmaceutical pairs. **Results:** ^18^F-FDG PET/CT demonstrated a high detection rate for distant metastases, outperforming conventional CT and MRI in advanced disease, despite limited resolution for infracentimetric nodal deposits. PET/MRI offers comparable diagnostic accuracy with superior soft-tissue contrast and improved brain lesion detection, while SPECT/CT enhanced sentinel lymph node localization prior to surgical biopsy. Also, FDG PET/CT identified visceral irAEs with great sensitivities, revealing asymptomatic toxicities in up to one-third of patients. Emerging radiotracers targeting melanin, fibroblast activation protein, PD-1 (programmed cell death protein 1)/PD-L1 (programmed cell death-ligand 1), and CD8^+^ T cells have demonstrated enhanced tumor specificity and are on their way to forming novel theranostic pairs. **Conclusions:** While ^18^F-FDG PET/CT remains the cornerstone of melanoma imaging, complementary advantages of PET/MRI and SPECT/CT imaging refine melanoma management. The advent of highly specific radiotracers and integrated theranostic approaches heralds a new era of tailored nuclear-medicine strategies, promising improved patient stratification, therapy guidance, and clinical outcomes in melanoma.

## 1. Introduction

Malignant melanoma continues to present significant diagnostic and therapeutic challenges. The prognosis depends strongly on stage at the time of diagnosis, localized disease (AJCC stages I and II) carries a good prognosis, regional node-positive disease (stage III) substantially increases the risk of relapse, and metastatic disease (stage IV) is associated with poor long-term survival, with a 5-year survival that drops dramatically to 15–20% in stage IV disease. The current AJCC (8th edition) TNM framework remains the reference for stage grouping and treatment decisions [1]. The emergence of targeted therapies and immune checkpoint inhibitors has improved outcomes, but optimal management heavily depends on accurate imaging to guide staging, selection of treatment, and monitoring patient response [2].

Three imaging paradigms commonly used in melanoma are PET/CT, PET/MRI, and SPECT/CT. The most popular, ^18^F-fluorodeoxyglucose PET/CT (FDG), combines positron emission tomography (functional, tracer-based imaging of metabolism or receptor expression) with high-resolution anatomic localization. FDG accumulates in glucose-hypermetabolic malignant cells, making it a sensitive modality for whole-body staging and recurrence detection. While conventional CT and MRI play important complementary roles, FDG PET/CT often reveals occult lesions and can identify disease earlier than conventional imaging [3]. However, limitations still persist: small pulmonary, hepatic, or brain metastases (<1 cm) may be missed, and high FDG-avid inflammation (e.g., immune-related adverse events) can complicate image interpretation [4]. PET/MRI couples the molecular sensitivity of PET with MRI’s superior soft-tissue contrast and multiparametric sequences; in melanoma, this can improve detection of brain, liver, and bone marrow lesions and reduce ionizing radiation exposure compared with PET/CT, though PET/MRI is less widely available and can miss small pulmonary nodules unless complemented by lung-optimized sequences [5,6]. SPECT/CT (single-photon emission computed tomography combined with CT) remains the standard technique for preoperative sentinel lymph node mapping. Lymphoscintigraphy with Tc-99m colloids plus SPECT/CT gives precise anatomic localization of sentinel nodes to guide surgical biopsy [7]. Each hybrid modality, therefore, has complementary strengths and limitations that influence how and when they are applied in staging algorithms.

Theranostics denotes the intentional pairing of a diagnostic radiopharmaceutical, used to image target expression, with a chemically related therapeutic radiopharmaceutical that delivers cytotoxic radiation to the same molecular target. This approach identifies patients whose tumors express a medication target, permits lesion-level dosimetry, and consequently directly informs the selection, planning, and predicted efficacy of radionuclide therapy. This integration of target identification, patient selection, and personalized dosimetry distinguishes theranostic strategies from traditional diagnostic imaging, which classically served staging and response assessment without enabling matched molecularly targeted radiotherapy [8,9].

There is growing interest in novel nuclear medicine theranostic techniques targeting melanin, fibroblast activation protein (FAP), or programmed cell death protein 1/programmed cell death-ligand 1 (PD-1/PD-L1). The latter modulates T-cell inhibition and is the target of the checkpoint inhibitors used in melanoma treatment. These new and exciting approaches to melanoma management could improve tumor diagnostic and treatment specificity while enabling theranostic strategies [10,11,12].

Early work on melanin-targeting iodinated benzamides demonstrated proof of principle for melanoma-selective radiotracers [13], and subsequent reviews [14] summarized early radiopharmaceutical options and lymphoscintigraphy approaches. More recent comprehensive efforts explored melanin-binding theranostic approaches in patients [15], and contemporary preclinical/translation studies (including 2022–2023 tracer development reports) have expanded targets to MC1R, FAP, PD-1/PD-L1, and CD8 imaging agents [16,17,18,19].

The present review updates and synthesizes prior contributions by placing them within the framework of contemporary hybrid imaging and radiopharmaceutical advances and by highlighting the practical implications of theranostic pairings for personalized management of malignant melanoma. In doing so, it summarizes current evidence on the clinical applications of radiopharmaceuticals across four main domains: diagnosis and staging; treatment response assessment and prognosis; detection of immune-related adverse events (irAEs); and emerging radiopharmaceuticals with future research directions.

## 2. Materials and Methods

This work was conducted as a narrative review because our primary objective was to synthesize a broad, multidisciplinary body of literature spanning molecular imaging, surgical oncology, dermatology, radiation oncology, and theranostics, and to produce a concise, clinically oriented guide that relates contemporary melanoma management to available and emerging molecular imaging techniques. A narrative approach allowed us to integrate heterogeneous evidence types (randomized studies, prospective and retrospective cohorts, small imaging series, technical reports, and preclinical tracer development), to highlight practical implications for clinicians, and to provide interpretive, practice-focused recommendations that would be difficult to capture within a more narrow question framing of other, more statistical techniques.

Focusing on PET/CT, PET/MRI, and SPECT/CT reflects the complementary and central roles these modalities play across the full clinical pathway of melanoma management. PET/CT provides established whole-body metabolic staging, quantitative biomarkers for prognostication and response assessment, and practical surveillance for immune-related toxicity. PET/MRI integrates PET’s metabolic sensitivity with MRI’s superior soft-tissue and central nervous system (CNS) resolution and additional functional MRI parameters, making it especially relevant where brain, liver, or marrow evaluation is critical. SPECT/CT (and lymphoscintigraphy) remains the reference technique for sentinel lymph-node localization and surgical guidance. Together, these platforms capture both the current clinical utility and the technological trajectories (novel PET tracers, immuno-PET, radiopharmaceuticals, theranostics) most likely to influence future practice.

Being narrative in nature, the review does not follow PRISMA or formal systematic review criteria, although it employs a focused literature search strategy. A literature search was conducted using PubMed, Scopus, and clinical-trial registries for original research articles, meta-analyses, systematic reviews, clinical guidelines, and illustrative case reports from January 2000 to January 2025. The Boolean string used was ((“melanoma”) AND (“PET/CT”) OR (PET/MRI) OR (SPECT/CT)). The search was limited to studies published in English. No formal MeSH terms or filters were applied because of the narrative perspective of this review, but preference was given to high-quality peer-reviewed studies, systematic reviews, and high-impact clinical research. A total of 305 potentially relevant publications were identified.

Sources included narrative reviews, systematic reviews, clinical studies, and case reports relevant to the nuclear medicine aspect of melanoma diagnosis and management. Publications such as letters to editors, conference abstracts, editorials, opinion articles, and unpublished material were excluded to maintain the relevance of this review.

**Figure 1:**
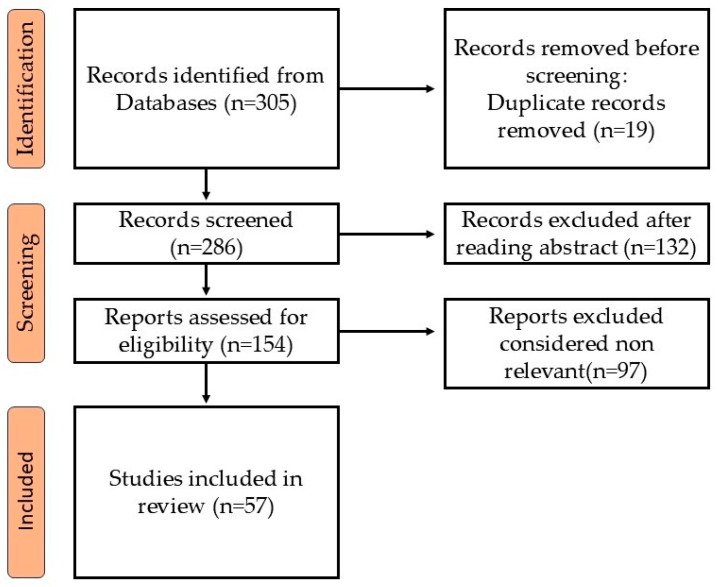
Flow diagram of the article selection process.

Figure 1 provides a summary of the literature search that was conducted and the selection process used. Some of the articles included in the study were not available in full-text–open access.

## 3. Results

### 3.1. Diagnostic and Staging Applications

Melanoma is an aggressive type of skin cancer with a very high metastatic potential. Determining whether the disease has spread to the lymph nodes or other organs is crucial for the management, prognosis, and treatment of this affliction. ^18^F-FDG PET/CT has become a key tool in melanoma management, showing a high diagnostic accuracy (pooled sensitivity 81% and specificity 92%) and a high sensitivity in detecting visceral and soft-tissue metastases (88% sensitivity, 94% specificity for distant disease) [20]. For initial staging of early melanomas (stages I-II), sensitivity is much lower, but ^18^F-FDG PET/CT excels in advanced cases, with a sensitivity reaching 90% [20,21].

According to the AJCC (8th edition), melanoma is classified via the TNM system as localized (stage I–II), node-positive (stage III), or advanced/metastatic (stage IV). Based on European consensus-based interdisciplinary guidelines, ^18^F-FDG PET/CT is recommended for the initial assessment of distant metastases in all patients with stage III or IV disease and in those with stage IIC melanoma who exhibit poor-prognostic features, and it is likewise indicated for routine follow-up of all stage IIC–IV melanoma patients [20]. Also, PET/CT plays a central role in monitoring the response to modern therapies (targeted agents, immunotherapy) and can detect immune-related adverse changes, having a “high diagnostic performance for detection of soft-tissue, nodal and visceral metastases” in melanoma [22]. Most centers obtain a dedicated MRI of the brain when its involvement is suspected. Modern PET/CT scanners (with low-dose CT) may obviate routine separate brain MRI, as studies have shown no additional brain mets on MRI when a PET/CT has been performed [2,23].

A meta-analysis of over 10,000 patients found a patient-level pooled 81% sensitivity and 92% specificity. ^18^F-FDG PET/CT detected distant metastases in approximately 88% of cases (specificity 94%) but detected regional lymph node metastases in only 56% of cases (specificity 97%) [20]. Therefore, it is an accurate instrument to rule in disease (with a high specificity and PPV) but misses a substantial fraction of small nodal metastases. At the lesion level, pooled PET/CT sensitivity was 70% (with a specificity of 94%), reflecting that infracentimetric disease can be occult, leading to a low sensitivity for microscopic nodal disease [20]. When melanoma is sufficiently metabolically active to show up on PET, PET/CT often detects metastases missed by CT alone. A Cochrane review found that PET/CT outperforms CT and MRI for detecting metastatic spread in melanoma. In patients with a recurrence, PET/CT proved to be more effective in detecting distant metastases than in primary staging [21].

### 3.2. Stage-Specific Performance

In early-stage (I-II) melanoma, ^18^F-FDG PET/CT has low utility, with a sensitivity often below 40% [20,23]. For example, in a prospecting staging of T2b-T4b melanomas, PET was relatively insensitive to small disease [20]. In contrast, in stage III (clinically node-positive or biopsy node-positive) and stage IV metastatic patients, PET/CT sensitivity rises. In one series of patients with stage III-IV melanoma being considered for surgery, PET/CT had approximately 86% sensitivity and 87% specificity for detecting deep nodal and visceral metastases [24]. Another meta-analysis of advanced cases reported a sensitivity of 86–90% and a specificity of 87–90%, making PET/CT an accurate tool for exposing apparent nodal disease or known metastases [20,24]. In practical terms, PET/CT is used at the initial work-up of high-risk stage II (Breslow > 2 mm with ulceration) and stage III melanoma, and always for stage IV disease. It is also used at the first relapse. By contrast, PET/CT is not indicated for routine staging of stage I-IIA disease without other risk factors (its low sensitivity yields little new information, as reflected by near-zero management change in some studies) [23,25]. In an Australian cohort of stage III-IV patients (with satellite/in-transit disease), whole-body PET/CT upstaged the disease and altered treatment in 16% of cases, confirming its impact in high-risk groups [23].

### 3.3. Comparison with Conventional Imaging

For distant staging (M staging), multiple studies show that PET/CT finds more metastases than a conventional CT or MRI. A meta-analysis found PET/CT to be better than CT in identifying spread in both primary and recurrent settings. In practical terms, PET/CT will often detect small visceral lesions that appear equivocal on CT. However, for regional nodal basin (N staging), ultrasound (often with a fine needle aspiration biopsy) remains more sensitive than PET/CT [21]. In one series, brain MRI did not find any metastases missed by PET/CT in baseline staging, leading some centers to omit a brain MRI if a PET/CT that includes the skull base is performed [23]. Most guidelines still recommend a brain MRI in advanced disease, but PET/CT can serve as useful brain screening when doing a whole-body acquisition [22,23].

### 3.4. PET/MRI in Melanoma

PET/MRI combines PET’s high sensitivity for metabolically active tumors with MRI’s superior soft-tissue contrast. Early data suggest PET/MRI can achieve a lesion detection performance comparable to PET/CT in melanoma. For example, Berzaczy et al. found that whole-body FDG-PET/MRI had an overall accuracy of 96% versus 97% for PET/CT in detecting melanoma metastases, with no significant difference (*p* = 0.42) [26]. This implies PET/MRI may match PET/CT in staging utility while offering added benefits. In particular, whole-body MRI is radiation-free and has “emerged as a competitive alternative to CT for staging advanced melanoma”. PET/MRI can potentially reduce cumulative radiation exposure (avoiding the CT component) and improve visualization of soft-tissue and brain lesions. Notably, MRI excels at identifying small asymptomatic brain metastases, which significantly impact prognosis in stage IV melanoma. MRI is “acknowledged superior” to CT for brain and spinal metastases [27]. This means PET/MRI can combine PET’s whole-body survey with high-resolution MRI of the brain or spine in one session, potentially catching small central nervous system mets missed by PET/CT.

However, PET/MRI is not sufficiently sensitive for microscopic nodal disease. In one series of 52 patients, FDG-PET/MRI (with or without diffusion-weighted MRI) identified only 4 of 17 histologically positive sentinel nodes (sensitivity approximately 24%), nearly identical to PET/CT. The authors concluded that integrated PET/MRI “does not reliably differentiate N-positive from N-negative melanoma” and cannot replace sentinel node biopsy [14].

PET/MRI’s MRI component may reveal subtle liver lesions or brain mets not visible on CT [27]. On the other hand, MRI has known limitations in lung imaging: multiple studies (and the above WB-MRI series) found whole-body MRI less sensitive than CT for detecting small pulmonary nodules. Conventional MRI sequences often miss infracentimetric lung mets due to motion artifacts. PET/MRI may require supplemental lung-focused imaging (e.g., a low-dose chest CT or advanced MRI sequences such as UTE) to avoid missing pulmonary metastases [28,29].

### 3.5. Sentinel Lymph Node Metastases Assessment (SLNM)

A key aspect of melanoma staging is the detection of the sentinel lymph node metastases (SLNM). Sentinel lymph node biopsy (SLNM): the first draining node(s) to be surgically removed and pathologically examined is the reference standard [30].

Single-photon SPECT/CT in melanoma is mainly used for sentinel lymph node mapping before surgical biopsy. A small dose of Tc-99m nanocolloid is injected near the tumor, and planar lymphoscintigraphy plus SPECT/CT is performed to locate all draining sentinel nodes. This hybrid approach offers precise anatomic localization and often identifies additional nodes missed on planar imaging. In one large surgical series, adding SPECT/CT to conventional planar mapping increased the average number of sentinel nodes identified (3 vs. 2) and detected more pathologically positive nodes (20.9% of SPECT/CT patients had positive SNs vs. 16.5% with planar only), concluding that SPECT/CT markedly increases the accuracy of sentinel node identification and is associated with better disease-free and disease-specific survival [7].

Multiple studies show PET/CT misses most microscopic nodal disease. For example, Kell et al. found that among early-stage patients undergoing SLNM, PET/CT identified only 2 of 9 positive sentinel nodes (PPV 24%, NPV 76%) [25]. In another study, nearly 46% of SLNM-positive patients had negative PET/CT within one year [31]. A negative PET/CT scan is very common, even when microscopic nodal metastases are present, showing that FDG-PET/CT has low sensitivity in finding regional nodal disease and cannot replace lymph node biopsy [20]. On the other hand, SPECT/CT and planar lymphoscintigraphy help surgeons locate sentinel nodes but do not characterize them as benign or malignant, whenever indicated (usually Breslow > 0.8–1 mm or other risk factors). If the sentinel node is positive on pathology, PET/CT is warranted to search for additional disease [7,32].

Table 1 presents a comparison between the three different diagnostic methods in melanoma, PET/CT, PT/MRI, and SPECT/CT, considering some of the most important features targeted by them.

## 4. Discussion

### 4.1. ^18^F-FDG PET/CT Demonstrated a High Detection Rate for Distant Metastases

Hybrid ^18^F-FDG PET/CT is well-established for staging advanced melanoma. It offers high sensitivity for systemic metastases. Meta-analyses report pooled sensitivities around 80–80% and specificities of about 85–95% for detecting non-nodal metastases [33,34]. These findings are reflected in clinical practice, with most guidelines now recommending PET/CT alongside a dedicated brain MRI for advanced melanoma stages [1,2,35]. ^18^F-FDG PET/CT often revealed occult metastases, changing the management of melanoma patients. In a series of 59 patients suspected of cutaneous melanoma, PET/CT detected more extensive disease in 55% of cases and altered the treatment for 49% of patients [36]. These studies underscore the high detection rate of PET/CT for clinically occult metastases in melanoma, often changing clinical management by upstaging patients or identifying candidates for systemic therapy. However, this technique is not without its limitations, the most significant of which is its poor sensitivity due to high normal FDG uptake in the brain tissue [37]. Small lesions in the lung can also be missed due to respiratory motion [33]. Similarly, ^18^F-FDG PET/CT performed with or without contrast showed equivalent accuracy in advanced melanoma, suggesting standard non-contrast PET/CT is usually sufficient [38].

### 4.2. PET/MRI Could Offer Comparable Diagnostic Accuracy

Studies comparing PET/MRI to PET/CT in melanoma and other cancers report similar accuracy. ^18^F- FDG PET/MRI appears to be comparable to PET/CT for lesion detection in malignant melanoma [26]. Broader systemic reviews across multiple cancers support this parity, showing no significant differences for nodal and metastatic lesions detected between the two [39]. In practice, this means PET/MRI can often substitute PET/CT without sacrificing accuracy [28,39,40]. However, PET/MRI scans generally take longer and are more expensive, which can limit availability. MRI is also less sensitive than CT for very small lung nodules or bone lesions. Conversely, PET/MRI excels in the assessment of the brain and liver. No large melanoma-specific trials have found PET/MRI inferior to PET/CT in sensitivity for extracranial metastases, but this remains an area of investigation. In practice, it seems that PET/MRI can replace PET/CT for whole-body staging without loss of accuracy, but CT may still be used if small pulmonary nodules or skeletal detail are critical.

### 4.3. SPECT/CT Enhances Sentinel Lymph Node Localization Prior to Surgical Biopsy

Accurate localization of sentinel lymph nodes (SLNs) is crucial in melanoma surgery. Preoperative lymphoscintigraphy traditionally uses planar images, but hybrid SPECT/CT has significantly improved sentinel node mapping. SPECT/CT provides a 3D localization, allowing surgeons to pinpoint SLSs in anatomically complex regions. Meta-analyses and large series have shown SPECT/CT outperforms planar imaging in SLN detection [41]. In practice, SPECT/CT has clarified ambiguous planar scans (e.g., overlapping hotspots) and reduced false negatives while also being associated with improved disease-free and disease-specific survival, likely by enabling more accurate staging and treatment. Also, SPECT/CT occasionally leads to more cancellations of SLN biopsy on the day of surgery, highlighting a trade-off: SPECT/CT can reveal advanced disease that changes management [7].

### 4.4. Emerging Radiotracers

Despite FDG’s utility, its lack of specificity spurs the development of novel melanoma tracers. A recent review identified four main classes in development: melanin-targeted, FAP-targeted, PD-1/PD-L1-targeted, and CD8^+^-T-cell tracers [42]. Melanin-targeted probes exploit the pigment abundant in many melanomas. For example, benzamide and acridine analogs labeled with ^18^F or ^11^C have been synthesized that bind melanin in tumors with high contrast. Early human studies of an ^18^F-labeled melanin ligand ([^18^F]DMPY2) demonstrated clear imaging of metastases. Likewise, ^123^I-labeled benzamide analogs have been used to find melanoma lesions in trials. These agents may detect small metastases (even brain or lung) that FDG misses, and they inherently target melanoma cells without binding inflammatory tissue [43].

Fibroblast activation protein inhibitors (FAPIs) are another emerging class. ^68^Ga-FAPI PET/CT targets cancer-associated fibroblasts and shows high uptake in many tumor types. Preliminary data suggest melanoma lesions can also concentrate FAPI tracers, reflecting the desmoplastic stroma of these tumors. FAPI imaging is still investigational in melanoma, but experts note its broad diagnostic potential [42].

Immuno-PET tracers are of special interest for therapy monitoring. Radiolabeled antibodies or fragments against PD-1, PD-L1, CTLA-4, or CD8 allow noninvasive mapping of immune checkpoint molecules or effector T-cells. For instance, ^18^F-BMS986192 (an anti-PD-L1 antibody fragment) and ^89^Zr-labeled anti-PD-1 antibodies have entered clinical trials in melanoma. These probes can quantify checkpoint expression and may predict or monitor response to immunotherapy. Similarly, ^89^Zr-atezolizumab (anti-PD-L1) imaging has shown that tumor uptake correlates with subsequent response. CD8-targeted PET probes (e.g., ^89^Zr-IAB22M2C) can image T-cell infiltration in tumors. While these tracers are not yet routine, they have “shown potential for response assessment and prediction” in early studies [42].

### 4.5. Detection of Immune-Related Adverse Events (irAEs)

Immune checkpoint inhibitors (ICIs, e.g., anti-PD-1/PD-L1) have markedly improved outcomes in melanoma management. However, ICIs frequently trigger immune-related adverse events (irAEs) affecting various organs. Dermatologic irAEs are most prevalent (occurring in up to ~50% of patients), and gastrointestinal (colitis) and endocrine (especially thyroiditis) toxicities are also more common. Severe grade ≥3 irAEs occur in roughly 14% of high-risk melanoma patients on adjuvant ICIs. Early detection of irAEs is critical to manage toxicity while maintaining effective therapy. ^18^F-FDG PET/CT is routinely used in melanoma for staging and monitoring. By imaging glucose metabolism, PET/CT not only detects cancer recurrence (sensitivity of about 90%) but also visualizes immune activation in normal tissues. In theory, this allows PET/CT to reveal sites of inflammation caused by ICIs. Gideonse et al. note that “^18^F-FDG-PET/CT can potentially be used to diagnose irAEs” by identifying immune-driven uptake before symptoms develop. Although not yet formalized in international guidelines, PET/CT surveillance in high-risk melanoma could therefore serve the dual purpose of tumor restaging and irAE screening [44].

Several studies have quantified PET/CT’s performance for irAE detection in melanoma. In a large retrospective series of 147 metastatic melanoma patients on ICIs, Murad et al. found that 36 (24.5%) developed clinically confirmed irAEs. PET/CT identified all 36 patients (100% sensitivity at the patient level) and detected 92% of individual irAE events. Remarkably, one-third (33%) of PET-detected irAEs had not been suspected clinically prior to imaging. The most common findings were diffuse thyroid FDG uptake (thyroiditis) and diffuse bowel uptake (colitis). The authors concluded that “^18^F-FDG PET/CT is highly effective in detecting irAEs in patients with metastatic melanoma”, uncovering asymptomatic toxicities in many cases [45].

Organ-specific accuracy varies. In an adjuvant melanoma cohort undergoing routine 3-month PET/CT scans, Gideonse et al. reported that 52.8% of patients developed irAEs in the first year. The highest PET sensitivity was seen for visceral irAEs: 100% for intestinal (colonic) inflammation, 92% for thyroiditis, and 71% for musculoskeletal (arthritis/myositis) events. Specificity was also good (85–95%) in these organs. By contrast, cutaneous irAEs were poorly detected (only 19% sensitivity), likely because inflammatory skin uptake can mimic melanoma lesions or postsurgical changes [44]. These findings mirror pooled data of a systematic review, which shows ^18^F-FDG PET/CT sensitivity ~89–100% for thyroiditis and 100% for colitis or pneumonitis in melanoma patients. Specificity is more variable, so PET findings must be interpreted with clinical correlation [46].

### 4.6. Therapy Response and Prognosis

^18^F-FDG PET/CT has an established role in advanced melanoma for staging and monitoring therapy. It provides functional data beyond anatomic scans, and PET-derived metrics (such as SUVmax, metabolic tumor volume (MTV), and total lesion glycolysis (TLG)) have been shown to add prognostic value in patients receiving targeted therapy or immune checkpoint inhibitors [33]. In recent years, attention has also turned to emerging PET tracers and radiopharmaceutical theranostics that more specifically target melanoma biology or the tumor microenvironment.

PET positivity generally indicates more aggressive biology: patients with higher baseline MTV or TLG tend to have shorter survival. For example, in BRAF-mutant melanoma treated with BRAF/MEK inhibitors, a baseline MTV above approximately 56 cm^3^ predicted worse progression-free survival (PFS). PET metabolic parameters also correlate with serum markers: high MTV correlates strongly with LDH and S-100 levels. Quantitative FDG uptake thresholds are sometimes used to risk-stratify patients before therapy. In one large meta-analysis, higher PET metabolic burden was consistently linked to poorer outcome in metastatic melanoma [33].

The PERCIST (PET Response Criteria in Solid Tumors) provides a standardized and quantitative framework for interpreting FDG PET/CT in oncology, enhancing reproducibility and prognostic relevance. PERCIST defines metabolic response based on the percent change in standardized uptake value normalized for lean body mass (SULpeak) of the most FDG-avid lesion, using ≥30% reduction (with ≥0.8 SUL units) as the threshold for partial metabolic response, and complete metabolic response requires FDG uptake in all lesions to fall below liver background levels without new lesions appearing. Baseline lesions must have uptake ≥1.5 times the mean liver SUL (plus 2 SD) to qualify for assessment. The criteria allow measurement of up to five lesions (maximum two per organ) and permit different lesions to be measured on follow-up scans, reducing interscan variability. This structured approach improves upon earlier EORTC PET response guidelines and is increasingly applied in solid tumors, including melanoma, to enable comparisons across studies and enhance the prognostic value of PET-based response assessment [47,48].

Because immunotherapies (e.g., anti-CTLA4, anti-PD-1) and targeted agents (e.g., BRAF/MEK inhibitors) have distinct response patterns, PET interpretation must be tailored. Early FDG PET after BRAF inhibitor therapy often reveals rapid decreases in tumor uptake in responders. Greater reductions in SUVmax or MTV on PET correlate with longer PFS and overall survival (OS). Complete metabolic response on PET (no FDG-avid disease) during targeted therapy predicts a very favorable prognosis [33].

Under immunotherapy, PET interpretation is more complex. Agents like ipilimumab and nivolumab can provoke immune-related inflammation (e.g., thyroiditis) that transiently increases FDG uptake in benign organs. For anti-CTLA4 therapy, an immune flare (new or worsening uptake due to immune cells) can confound early scans. In practice, criteria such as EORTC, PERCIST, or immunotherapy-modified PERCIST (imPERCIST) have been applied. While EORTC/PERCIST criteria often correlate with outcomes, they may misclassify early immunotherapy flare. Alternative metrics like PERCIMT (counting new lesions) or combining PET metrics (MTV, TLG) with EORTC can improve accuracy. For example, one study found that combining MTV and EORTC best predicted response to anti-PD-1 therapy, whereas PERCIMT plus MTV was superior for anti-CTLA4 therapy [48,49,50].

Crucially, metabolic response on PET strongly predicts clinical outcome. A decrease in whole-body MTV after several cycles of therapy is associated with prolonged OS. Patients achieving a complete metabolic response (CMR) by PET have markedly higher long-term survival. In one cohort, 5-year PFS was significantly higher in patients with CMR than in those with any residual uptake [51]. Even after treatment discontinuation, the absence of FDG-avid lesions portends excellent outcomes. These data underscore that PET-derived response metrics outperform anatomic criteria for predicting prognosis in treated melanoma.

PET/MRI may also influence therapy monitoring and clinical management. FDG-PET/CT is known to guide melanoma treatment, but PET/MRI offers additional functional insights. In melanoma models, both FDG-PET and diffusion-weighted MRI have proven useful as non-invasive biomarkers of response to targeted therapy [52]. For example, decreases in tumor FDG uptake and increases in ADC on DWI correlated with tumor cell kill after combination targeted treatment. By capturing both measures simultaneously, PET/MRI could potentially stratify responders early in therapy. Clinically, PET/MRI has altered management in case reports. One illustrative case showed that PET/MRI detected multiple small liver metastases that PET/CT missed. In that 59-year-old patient, the FDG-PET images were unremarkable on both PET/CT and PET/MRI, but the contrast-enhanced MRI component of PET/MRI revealed new hepatic lesions. These findings were not seen on the CT from PET/CT. The PET/MRI discovery changed the diagnosis to progressive disease and prompted a switch from ipilimumab to anti-PD-1 therapy. This example underscores that PET/MRI can impact management when its MRI data add information [27]. More generally, by integrating metabolic and functional MRI data (for example, combining FDG uptake, ADC mapping, or other MRI biomarkers), PET/MRI may help distinguish tumor progression, pseudoprogression, or immune-related changes during immunotherapy, although formal studies in melanoma are still limited.

### 4.7. Theranostics in Melanoma

Theranostics, the pairing of diagnostic imaging with targeted radionuclide therapy, is an emerging field in medical oncology. In melanoma, no FDA-approved theranostic is yet standard, but several strategies are under investigation.

Melanin is abundant in many melanoma cells, so radiolabeled small molecules that bind melanin have been explored for both imaging and radionuclide therapy. For instance, ^123^I-BZA2 (N-(2-diethylaminoethyl)-2-iodobenzamide) was tested in a multicenter trial to image metastases by SPECT. However, its sensitivity was low (~39% patient-based) because many metastases lack dense pigment. In that trial, only 45% of biopsied metastases were melanin-rich, and ^123^I-BZA2 detected most of those but missed many amelanotic lesions. Nonetheless, when lesions are pigmented, these tracers can show long tumor retention and high contrast [53].

Newer melanin-targeted PET tracers have shown better performance. For example, ^18^F-PFPN (a fluorinated picolinamide) binds melanin with high affinity; in a pilot clinical study, it was safe and able to outperform ^18^F-FDG. PFPN showed higher uptake than FDG in primary tumors and nodes, yielding a higher lesion detection rate. These results demonstrate that melanin-specific PET can significantly improve sensitivity when melanoma cells contain pigment [54].

Some melanin-binding probes serve as theranostic pairs when labeled with a therapeutic radionuclide. For example, ^131^I-BA52 (a 131I-labeled benzamide similar to BZA2) was given to a cohort of metastatic melanoma patients after diagnostic imaging. Dosimetry showed that melanoma nodules could receive approximately 12 Gy per GBq of ^131^I-BA52, indicating potential for more aggressive treatment in future trials [15]. Similarly, ^131^I-ICF01012 (N-(2-diethylaminoethyl)-6-iodoquinoxaline-2-carboxamide), a new melanin-binding compound, is under phase I clinical evaluation (MELRIV-1) for targeted radionuclide therapy [55].

Also, melanin-binding monoclonal antibodies (c8C3) have been labeled with 203Pb (for SPECT) and ^212^Pb (alpha-particle emitter) as a matched pair. In melanoma-bearing mice, ^203^Pb-c8C3 imaged tumors on SPECT/CT and ^212^Pb-c8C3 delivered cytotoxic alpha radiation, leading to tumor suppression. This ^203^Pb/^212^Pb pair exemplifies a true theranostic system for melanoma [56].

Another strategy exploits the copper transporter CTR1, which is overexpressed in melanoma. The inorganic salt ^64^CuCl2 has been tested as a theranostic approach that images CTR1-positive tumors on PET and also delivers therapeutic radiation. Preclinical studies showed that ^64^CuCl2 PET clearly visualized both pigmented and amelanotic melanoma xenografts, and systemic ^64^CuCl2 therapy significantly slowed tumor growth compared to controls. So, copper isotopes (^64^Cu for PET and potentially ^67^Cu for therapy) represent a simple theranostic pair for melanoma with ubiquitous target expression [57].

Melanocyte-stimulating hormone (α-MSH) analogs targeting MC1R represent another theranostic avenue. In a first-in-human PET study, ^68^Ga-DOTA-GGNle-CycMSH (a cyclic Nle-modified α-MSH peptide) enabled clear visualization of melanoma metastases, including brain lesions. This study confirmed that MC1R is a viable clinical imaging target and suggests that MC1R-targeted radionuclide therapy (e.g., with ^177^Lu-labeled MSH analogs) could be feasible [58]. Next, ^177^Lu- or ^225^Ac-labeled cyclic α-MSH peptides have achieved tumor targeting in animal models. One study reports a “metabolically stabilized” ^177^Lu–MC1R ligand that extended survival in treated mice. Likewise, novel terbium-155/161 (^155^Tb/^161^Tb)-labeled α-MSH analogs have been prepared for combined PET/SPECT imaging and beta therapy in preclinical models. These MC1R-directed radioligands could enable both imaging (with ^68^Ga, ^64^Cu, ^89^Zr etc.) and therapy (with ^177^Lu, ^225^Ac, ^161^Tb) of metastatic melanoma. Reviews have noted that MC1R- and melanin-based probes “demonstrated promising and valid melanoma theranostic strategies” [43,59,60].

Other peptide or small-molecule theranostics include FAP-targeting agents. Although mostly studied in other cancers, FAP-targeted radioligand therapy (e.g., ^177^Lu-FAPi) could theoretically be applied in melanoma stroma-rich lesions. Additionally, bispecific or single-domain antibody fragments against tumor antigens (e.g., GD2, CSPG4) are being explored for radiolabeling with ^177^Lu or ^89^Zr to deliver targeted radiotherapy [59].

### 4.8. Limitations of the Study

This narrative review has several limitations. It is not a formal systematic review, so selection and reporting biases are possible. Many cited studies are retrospective or single-center series with heterogeneous patient populations and imaging protocols. There is no quantitative meta-analysis of PET/MRI or SPECT/CT performance due to limited data. Our discussion of novel tracers is also preliminary, based on early-phase trials and small cohorts. Finally, new therapeutic advances (e.g., adjuvant checkpoint inhibitors, targeted agents) are rapidly evolving, and the role of imaging in these contexts continues to be defined. Therefore, the conclusions should be interpreted with caution, pending further prospective validation.

## 5. Conclusions and Recommendations

^18^F-FDG PET/CT is established as a cornerstone in advanced melanoma management, with high accuracy for detecting distant metastases and guiding systemic therapy decisions. PET/MRI provides similar staging value, with the benefit of enhanced soft-tissue contrast and no extra radiation, making it useful in select cases. SPECT/CT remains indispensable for precise sentinel node mapping. Importantly, routine PET/CT scans can simultaneously reveal immune-related adverse events at early stages. Emerging PET tracers targeting melanin, PD-1/PD-L1, FAP, and CD8 show promise for even more specific imaging and may improve lesion detection or predict immunotherapy response. In practice, the key take-home messages are that FDG-PET/CT excels at whole-body staging in high-risk melanoma; PET/MRI could be an equivalent alternative when available; SPECT/CT enhances sentinel-node surgery planning; and novel radiotracers are under active investigation but not yet in routine use. As molecular imaging technologies and targeted therapies advance, integrating these tools thoughtfully will further improve personalized care and outcomes for melanoma patients.

## Figures and Tables

**Table 1:** Comparison of PET/CT, PET/MRI, and SPECT/CT in melanoma.

Feature	PET/CT (Mostly ^18^F-FDG)	PET/MRI (Mostly ^18^F-FDG)	SPECT/CT (Tc-99m Colloids, Others)
Primary tracer(s)	^18^F-FDG (commonly) emerging PET tracers (melanin, FAPI, PD-1/PD-L1, CD8, etc.)	^18^F-FDG and emerging PET tracers (same as PET/CT)	Tc-99m nanocolloid for lymphoscintigraphy; ^123^I-melanin agents historically for SPECT
Main strengths	High whole-body sensitivity for distant/visceral metastases quantitative metrics (SUV, MTV, TLG). Established, widely available.	Comparable whole-body accuracy to PET/CT with superior soft-tissue contrast (brain, liver, bone marrow) and lower ionizing dose (no CT). Good multiparametric MRI data (DWI, ADC).	Excellent anatomic localization of sentinel draining nodes improves planar lymphoscintigraphy with 3D localization. Well established for SLN mapping.
Best clinical uses in melanoma	Initial staging/restaging in stage III–IV and selected high-risk stage II (IIC) detection of occult distant metastases; therapy response/prognosis and irAE detection.	Selected staging where brain/liver/bone detail matters (e.g., high risk for CNS disease), therapy monitoring when MRI biomarkers add value, or when radiation dose reduction is desired.	Preoperative sentinel lymph node mapping to guide biopsy/surgery, clarifying ambiguous planar images and surgical planning in anatomically complex drainage.
Sensitivity/specificity	Patient-level pooled 81% sensitivity, 92% specificity for FDG PET across melanoma cohorts; higher (86–90%) in advanced disease for distant mets; lower for microscopic nodal disease.	Lesion-detection accuracy comparable to PET/CT in reported series; similarly low sensitivity for microscopic sentinel nodes.	Increases sentinel node detection vs. planar imaging; does not determine node malignancy.
Detection of microscopic/sub-cm nodal disease	Limited, low sensitivity for micrometastases. It cannot replace sentinel node biopsy.	Same limitation as PET/CT; not a reliable substitute for SLN biopsy.	Designed for mapping sentinel nodes (high anatomic localization), but cannot characterize metastatic involvement (pathology still required).
Brain metastases	FDG PET has limitations in brain (high background). Brain MRI often recommended in addition for dedicated CNS staging.	Superior, PET/MRI combines PET with high-resolution brain MRI, improving small CNS metastasis detection in one session.	Not used for brain metastases assessment.
Lung/small pulmonary nodules	Good for many pulmonary mets, but small infracentimetric nodules can be missed (respiratory motion).	Less sensitive than CT for very small lung nodules unless complemented by lung-optimized MRI or supplemental chest CT.	Not used for pulmonary staging.
Immune-related adverse events (irAEs)	High sensitivity for many visceral irAEs (thyroiditis, colitis, pneumonitis) and can reveal asymptomatic irAEs and influence management.	Can detect irAEs if PET tracer shows uptake; MRI component may add organ detail, but PET/CT is the more commonly used modality for irAE surveillance.	Not routinely used for irAE detection.
Therapy response/prognostic utility	Strong; metabolic metrics (SUV, MTV, TLG) correlate with response, and prognosis used with PERCIST/EORTC/imPERCIST frameworks.	Comparable for metabolic assessment; adds MRI biomarkers (ADC, perfusion) that can help distinguish pseudoprogression vs. true progression in some cases.	Not used for quantitative systemic therapy response assessment.
Theranostic potential	High; PET tracers (melanin, MC1R, FAPI, PD-1/PD-L1, CD8, ^64^/^67^Cu pairs, ^89^Zr immuno-PET) are central to future theranostic strategies.	Same PET tracers possible; PET/MRI can be used for patient selection/dosimetry where MRI soft-tissue detail matters.	Limited theranostic role; some melanin SPECT agents (^123^I) that historically used diagnostically therapeutic radionuclide pairs have been developed, mainly in PET/therapy radionuclide contexts.
Unique value/impact on management	Often uncovers occult distant disease and changes management (upstaging, treatment selection) established for response/irAE monitoring.	Replaces PET/CT in selected patients where brain or soft-tissue detail or radiation reduction matters can change management when MRI adds lesions missed on CT.	Directly guides sentinel node biopsy, improves surgical planning, and may detect additional sentinel nodes and reduce intraoperative uncertainty.

SLN = sentinel lymph node; irAE = immune-related adverse event; SUV = standardized uptake value; MTV = metabolic tumor volume; TLG = total lesion glycolysis.

## Data Availability

Data supporting the reported results can be found using public scientific databases. The authors confirm that the data supporting the findings of this study are available within the article.
